# Complex Visual Search in Children and Adolescents: Effects of Age and Performance on fMRI Activation

**DOI:** 10.1371/journal.pone.0085168

**Published:** 2013-12-23

**Authors:** Karen Lidzba, Kathina Ebner, Till-Karsten Hauser, Marko Wilke

**Affiliations:** 1 Pediatric Neurology and Developmental Medicine and Experimental Pediatric Neuroimaging, Children's Hospital University of Tübingen, Tübingen, Germany; 2 Diagnostic and Interventional Neuroradiology, Radiological Clinic, University of Tübingen, Tübingen, Germany; Universiteit Gent, Belgium

## Abstract

Complex visuospatial processing relies on distributed neural networks involving occipital, parietal and frontal brain regions. Effects of physiological maturation (during normal brain development) and proficiency on tasks requiring complex visuospatial processing have not yet been studied extensively, as they are almost invariably interrelated. We therefore aimed at dissociating the effects of age and performance on functional MRI (fMRI) activation in a complex visual search task. In our cross-sectional study, healthy children and adolescents (n = 43, 19 females, 7-17 years) performed a complex visual search task during fMRI. Resulting activation was analysed with regard to the differential effects of age and performance. Our results are compatible with an increase in the neural network's efficacy with age: within occipital and parietal cortex, the core regions of the visual exploration network, activation increased with age, and more so in the right than in the left hemisphere. Further, activation outside the visual search network decreased with age, mainly in left inferior frontal, middle temporal, and inferior parietal cortex. High-performers had stronger activation in right superior parietal cortex, suggesting a more mature visual search network. We could not see effects of age or performance in frontal cortex. Our results show that effects of physiological maturation and effects of performance, while usually intertwined, can be successfully disentangled and investigated using fMRI in children and adolescents.

## Introduction

Visuospatial processing encompasses a multitude of functions with varying degrees of complexity, such as visual perception, visuospatial attention, object categorization, spatial transformation, occulomotor planning and execution, etc. [1]. Given the broad range of subfunctions which are involved when processing complex visuospatial information, it is not surprising that widely-distributed brain networks are employed to manage these processes. 

### 1.1: Visual Search and Visual Exploration

In the present experiment we studied complex visual search, i.e., the wilful exploration of a complex visual stimulus in search for changing targets. This task demands both basic and complex search processes. The successful manipulation of complex visuospatial information is primarily dependent on occipito-temporo-parietal structures, which are commonly accepted to constitute the ventral and dorsal pathways of visual processing [1,2]. 

#### 1.1.1: Basic visual search

Classic visual search paradigms usually require the search for a predefined target in an array of distractors, where subjects perform overt or covert shifts of attention or gaze towards simple visual stimuli (e.g., 3-7). When shifting either attention or gaze, the frontal eye fields (FEF), situated within the posterior superior frontal gyrus (SFG) [8] are invariably activated [9,10-12]. The top-down control of visuo-spatial attention [9,13-18] and the representation of spatial coordinates [19] on the other hand, have been associated with bilateral superior parietal lobule (SPL) and intraparietal sulcus (IPS) in fMRI studies using a number of basic visual search tasks. 

#### 1.1.2: Complex visual search

The conscious and planned shifting of attention focus is one of the functions most relevant for the more complex task of visual exploration [20,21]. Only few studies have examined visual search under natural conditions, i.e., the free exploration of a complex visual scenery. With such a paradigm, Himmelbach and colleagues [22] identified the posterior parietal cortex, IPS, FEF, the insula, the temporo-parietal junction, and STG/STS as areas involved in natural visual search. Complex visual search tasks may, in addition, involve prefrontal networks including superior frontal sulcus (SFS) or ventrolateral / dorsolateral prefrontal cortex. This involvement has been interpreted to reflect a visual working memory component [15,23-25]. 

From lesion studies, it is well known that visual exploration relies on a predominantly right-hemispheric and densely interconnected perisylvian network (i.e., superior / middle temporal, inferior parietal, and ventrolateral prefrontal cortex of the right hemisphere, including the corresponding fibre-tracts). Disruptions of this network lead to the core symptoms of spatial neglect (i.e., contra-lesionally biased gaze orientation and visual exploration [26]). With the clear preponderance of right-hemispheric lesions leading to spatial neglect, it is somewhat puzzling that studies with healthy participants rarely find a strong lateralization of the above-mentioned functions. This may be related to task demand, since task complexity or increased processing demands were associated with a more bilateral activation pattern in complex visual search [27,28] and in mental rotation [29]. The Embedded Figures Task (EFT) can be considered another example of a more complex visual search task. Here, subjects are asked to search a complex figure for a simple geometric shape. The theoretical focus of the EFT lies on the assumption that local details have to be “disembedded” from the global gestalt of the stimulus [30]. Similarly to basic and complex visual search tasks [9,13-18], however, fMRI studies with the EFT highlight a bilateral parieto-occipital network comprising mainly SPL and precuneus [31]. 

### 1.2: Development of Visuospatial Functions

It is well known that - like most cognitive abilities - performance in visuospatial tasks improves during childhood and adolescence, i.e., as a function of normal brain maturation (e.g., visuospatial attention [32]; visuospatial analysis [33]; visuospatial memory [34]). However, the underlying neuronal mechanisms are not yet entirely clear. 

#### 1.2.1: Developmental changes in task-related fMRI activation

In the last decade, an increasing number of fMRI studies have provided evidence for two trends: On the one hand, brain regions may become more functionally specialized with age (for reviews, see 35,36). On the other hand, a “frontalization” of activation seems to reflect the ongoing maturation of the frontal cortex [37]. 

Functional specialization can be inferred from an increase in focus and (in some cases) in lateralization in older as compared to younger children [35,36]. Such a shift from diffuse to focal activation with increasing age is thought to be caused by the combination of an activation increase in task-critical areas and a decrease of activation in areas less relevant for the task [35]. However, unspecific changes in vascular coupling or in data quality (e.g., movement artefacts) may have to be taken into account when interpreting age-related localization of fMRI activation [38]. 

Apart from maturation of the relevant neural networks, the variation in activation patterns between children and adults may also be determined by the cognitive strategies employed [35]. For example, children often have more left-hemispheric activation than adults in fMRI tasks on mental rotation, which has been explained by more piecemeal-like vs. holistic strategies employed by the two groups [39]. In general, however, as children and adolescents mature, cognitive processing increasingly depends on top-down control: Inhibitory control increases and the underlying networks become more efficient and focussed. Thus, task-relevant regions increase in activation, while task-irrelevant regions demonstrate an activation decrease the older the individual gets [40,41]. In addition, the mostly prefrontally represented executive functions, responsible for efficacy and precision, become increasingly available. This can be suspected to be an important explanatory factor for age- and performance related differences in fMRI activation patterns observable during complex visuospatial tasks. 

#### 1.2.2: Effects of age and performance in visuospatial tasks

The fronto-striato-parieto-temporal network involved in the allocation of visuospatial attention matures progressively with age [42]. For complex visual search, increasing right-hemispheric lateralization with increasing age has been demonstrated by fMRI during late childhood and adolescence [28]. In the embedded figures test (EFT), the activation of typically developing adolescents did not differ from that of adults [43,44], but children demonstrated more left-hemispheric prefrontal activation. This was assumed to reflect verbal strategies and increased effort [45]. Data regarding visuospatial working memory is less clear. While in fMRI, an age-related shift from diffuse to focal activation within the core network has been observed [24,46-48], there were no age-related changes in a study using functional transcranial Doppler sonography [49]. Since performance gains are almost invariably related to normal development, it is both important and difficult to disentangle the two components in studies of age-effects on fMRI activation. The few studies making an attempt to meet this statistical challenge detected either no performance effects (e.g., 50-53), or a performance-related activation-increase in task-related brain regions which was independent of age (e.g., 42,54). 

With this study, we planned to assess the effects of both physiological maturation and performance in healthy children and adolescents during a complex visual search task, i.e., during the wilful exploration of a complex visual stimulus in search of a changing target. Based on the literature outlined above, we expect (H1) an fMRI activation increase within and an activation decrease outside the core visual attention network (i.e., right SPL, IPS, and FEF) with increasing age, reflecting a focussing of activation; (H2) an age-independent increase of lateralization with increasing performance within the same regions; and (H3) an increase of fMRI activation with age and performance in prefrontal cortex. 

## Materials and Methods

### 2.1: Participants

Fourty-three neurologically healthy children and adolescents (24 male, 19 female; mean age 12 years; range 7 to 17 years; native German speakers) participated in this study. According to the Edinburgh Handedness Inventory [55], 38 subjects were right-handed, 4 were left-handed, and 1 ambidextrous. Exclusion criteria were common MR-contraindications, prematurity and pre-existing neurological or psychiatric disorders. Data of three subjects was excluded due to technical problems in the recording of performance data, and one further subject (11 year old boy) was excluded due to extremely low performance (hit rate more than 2 standard deviations below the mean). All included subjects completed the German adaptation of the Wechsler Intelligence Scale for Children (HAWIK-IV) to ensure a normal level of cognitive abilities (mean full scale IQ = 110.02, SD = 8.02). 

#### 2.1.1: Ethics Statement

The study was approved by the ethics committee of the Medical Faculty of the University of Tübingen. All participants and their parents gave written informed consent. Subjects were compensated for their participation according to the time they spent on the study. 

### 2.2: Complex Visual Search Task

**Figure 1:**
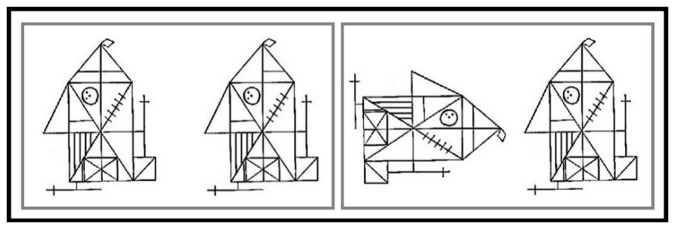
Examples for search condition (left) and control condition (right) of the complex visual search task.

The task is described in detail in [56]. In summary, subjects were presented pairs of abstract images derived from the Rey complex Figure [57]. In the search condition, 50% of the pairs were identical, while in the other trials a detail was missing in one of the figures (Figure 1, left). In the control condition, both figures were identical in all cases, but in 50% one of the figures was rotated (Figure 1, right). Stimuli were presented for 6 seconds each. Six control blocks alternated with 5 search blocks (each containing 5 stimuli), leading to a total task duration of 5:30 min. The task was practiced outside the scanner, and subjects were reminded of the task instructions directly before the start of the scan by a short video. The beginning of each new block was indicated by the short presentation of a blank green screen. Participants were told to press a button whenever the two figures were not the same, i.e., when a detail was missing (search condition) *or* when the orientations were different (control condition). They were explicitly told that the search condition was more difficult than the control condition and that they were to keep searching until they either had found a target (i.e., a difference between the figures), or until the next stimulus was presented. We assumed that the search condition differentially engages visuospatial working memory (keeping the "correct" pattern online), top-down guided visual attention shifts (systematically searching the patterns), saccades (between the two versions and within the searched pattern), pattern recognition (comparing the details of the searched pattern), and inhibition (e.g., of already searched parts of the pattern). Hits and misses during target trials as well as mean reaction time for hits were recorded. For further analyses, we defined hit rate as measure of performance and applied a z‑transformation, leading to positive z‑scores for relatively higher hit rates and to negative z-scores for relatively lower hit rates. 

### 2.3: Data acquisition and processing

Data was acquired on a 1.5-T whole body MR scanner (Avanto, Siemens Medizintechnik, Erlangen, Germany), using a 12-channel head coil. Care was taken to ensure comfortable placement of the participants, and a foam cushion was used to minimize head movement. Visual stimulus delivery was achieved by screen projection, using a custom-made MR compatible setup as used before [27]. A single MR-compatible pushbutton (Current Design Inc. Philadelphia, PA, USA) held in the left hand (by all subjects) was used for performance monitoring. For stimulus presentation and recording of button presses we used the *Presentation* software package (version 14.8, Neurobehavioral systems, Inc., Albany, CA). 

A T2-weighted echo-planar imaging (EPI) sequence was used to acquire functional images with the following parameters: Repetition time (TR) = 3000 ms, Echo time (TE) = 40 ms, matrix = 64 x 64, 40 slices, no interslice gap, covering the whole brain with a voxel size = 3 x 3 x 3 mm. For each task, 110 volumes were acquired. Additionally, a gradient-echo B0 fieldmap was acquired with TR = 546 ms, TE = 5.19/9.95 ms, with the same slice prescription as the functional series. For preprocessing purposes, we also acquired a T1-weighted 3D-data set with TR = 1300ms, TE = 2.92 ms, yielding 176 contiguous slices with an in-plane matrix of 256 x 256, resulting in a voxel size of 1 x 1 x 1 mm. 

Data was processed using SPM8 software (Wellcome Trust Centre for Neuroimaging, London, UK), running in Matlab (MathWorks, Natick, MA, USA). The first 10 scans of each functional series (corresponding to the first block of the control condition) were rejected to allow for the stabilization of longitudinal magnetization, leaving 100 scans per series (5 blocks each of the active and the control condition) to be analyzed. Functional images were realigned and unwarped using the individually-acquired B0 fieldmap, correcting for both EPI and motion*B0 distortions [58]. Functional series with translation exceeding one voxel size (3 mm) in any direction were discarded. The anatomical dataset was segmented using unified segmentation [59], based on custom-generated paediatric reference data [60]. Following coregistration of functional and anatomical data, these parameters were used to normalize the functional images. Global signal trends were removed [61] and functional images were smoothed with a 9 mm full width at half maximum (FWHM) Gaussian filter.

### 2.4: Statistical analyses

Functional MRI data was analysed on the first level in the framework of the General Linear Model (GLM) [62], contrasting the search condition with the control condition, including individual motion parameters as nuisance variables [63]. 

To test our hypotheses, we took a hierarchical approach. First, we determined the global task-related network by assessing the main effect of a whole-brain one-sample t-test (second level analysis), treating hit rate and age as covariates of no interest. Gender did not demonstrate any effects on performance (see results), so this variable was left out of the analysis. Statistical significance was assumed at an FWE-corrected p < .05 and an extent-threshold of k = 20. 

The first two hypotheses (H1 and H2) were then addressed by exploring the effects of age and performance within and outside this global task-related network. To this effect, we created a region of interest (ROI) from the global activation cluster of the main effect (thresholded at FWE-corrected p<.05; this ROI will be referred to as Global_FWE_). Global_FWE_ was used once as an inclusive and once as an exclusive mask for Analyses of Covariance (ANCOVA), with age and performance as covariates of interest. Since age and hit rates were correlated (see results), we orthogonalized the covariates onto each other for these analyses. For the ROI analyses, significance was assumed at *p* < .001 (uncorrected) and an extent cluster of k = 15. Lateralization was assessed using the LI-toolbox [64], based on mean individual activation levels within and outside Global_FWE_. In order to exclude a bias due to different search volumes in the two hemispheres, a symmetrical version of Global_FWE_ was used, by combining the original ROI with its flipped version. To avoid the threshold dependency of simple lateralization indices, a bootstrapping approach was employed. This approach analyzes a multitude of bootstrapped resamples from the original dataset at different thresholds and determines a weighted mean LI [65]. Positive LIs represent predominantly left-hemispheric activation, negative LIs represent a right-hemispheric preponderance [64]. 

In a third step, we explored the specific effects of age and performance within the frontal lobes (addressing H3). To this effect, we conducted an ANCOVA with age and performance (orthogonalized) as covariates of interest in an anatomically defined ROI (frontal cortex and insula plus prefrontal portions of cingulate and corpus callosum, according to the Hammersmith atlas [66]). Again, significance was assumed at *p* < 0.001 (uncorrected) and an extent cluster of k = 15. 

## Results

### 3.1: Behavioural Data

Hit rate (HR) of all participants was moderate during the search condition (mean 47% correct, SD = 15, range 25% - 83%), mean hit reaction time (RT) was 3133 ms (SD = 744). As expected, the control condition was much easier to solve (mean 93% correct, SD = 23, range 45% - 100%; mean hit RT: 1483 ms [SD = 753]). Age was correlated significantly with hit rate (*r*(36) = .434, *p* = .003), but not with RT (*r*(36) = .059, *p* = .361) in the search condition. Gender did not significantly influence performance in the search condition (RT: mean_males_ = 3162 ms [SD = 821], mean_females_ = 3095 ms [SD = 654], *t*(37) = -.276, *p* = .784; HR: mean_males_ = 47.45% [SD = 15.70], mean_females_ = 46.76% [SD = 13.56], *t*(37) = -.144, *p* = .886; two-sample *t*-tests). 

### 3.2: Global effects

All functional time-series with complete performance data were kept for analysis as none of the subjects displayed motion exceeding 3 mm in any direction. 

**Figure 2:**
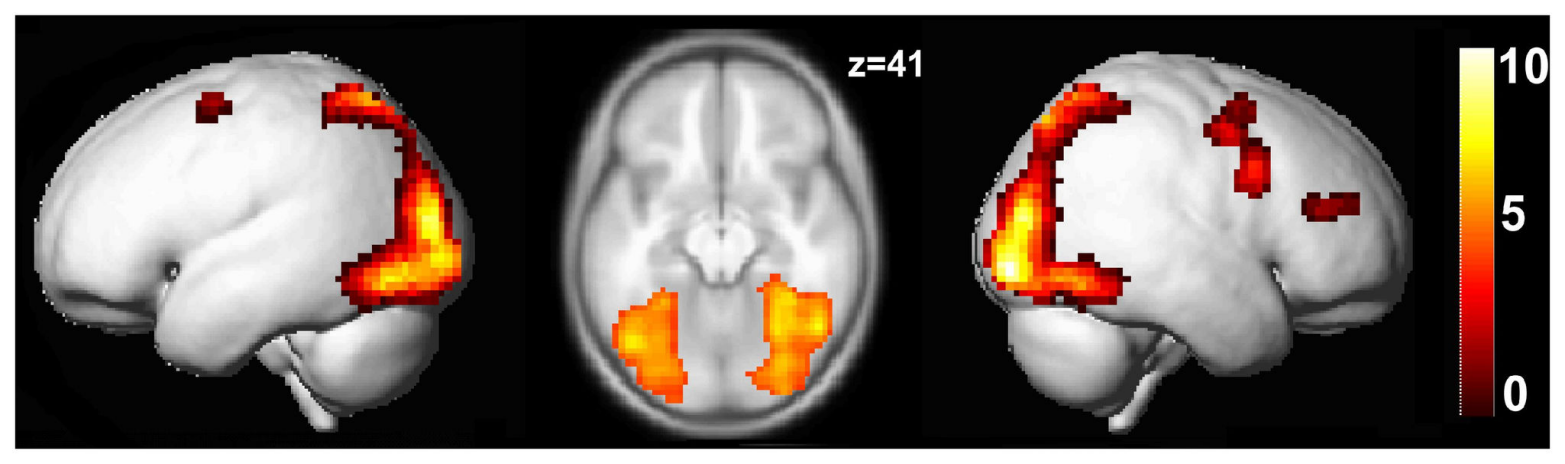
fMRI group activation at p < .05, FWE-corrected threshold for complex visual search vs. control and extent threshold k > 20.

On the group level, the search vs. control contrast revealed significant bilateral activation which included occipital cortex as well as inferior temporal gyri and bilateral, but right lateralized superior parietal cortex. In addition, a large cluster in the right and a smaller cluster in the left premotor cortex (middle frontal gyrus and precentral gyrus) were significantly more active in the search than in the control condition (Figure 2). 

### 3.3: Age effects

**Figure 3:**
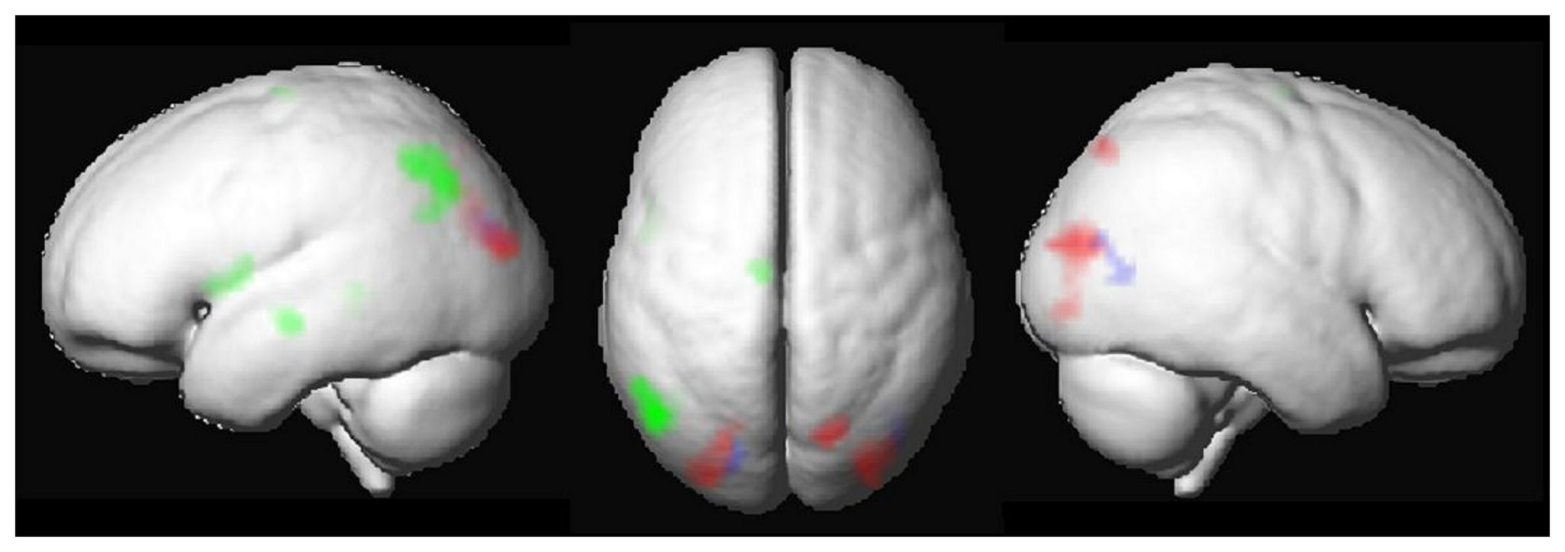
Age-related activation increases within (red) and outside (blue) the global task-related network (Global_FWE_). No age-related activation decreases were detected within Global_FWE_. Age-related activation decreases outside Global_FWE_ are depicted in green. All clusters within ROIs at uncorrected p < .001 and extent threshold k > 15.

The ROI analysis revealed age effects within and outside Global_FWE_ (Figure 3, Table 1). Inside the ROI, age correlated positively with activation in both lateral occipital lobes and at trend-level in right superior parietal lobe. Outside the ROI, a marginal positive effect of age was detected in both occipital lobes, extending the clusters within Global_FWE_. Within Global_FWE_, no significant negative correlations with age were detected. However, outside the ROI, we detected a significant negative effect of age in left inferior parietal lobe, left middle temporal gyrus, and left inferior frontal gyrus.

**Table 1:** Age-related fMRI activation.

	Inside Global_FWE_				Outside Global_FWE_			
	Peak level		Cluster level		Peak level		Cluster level	
	T	p_uncorr_	k_E_	p_uncorr_	T	p_uncorr_	k_E_	p_uncorr_
	**Positive** effect of age							
right lateral occipital cortex	4.93	<.0001	105	.012	3.93	<.0001	23	.194
left lateral occipital cortex	4.67	<.0001	109	.004	4.50	<.0001	26	.147
right superior parietal lobule	4.47	<.0001	30	.110	-	-	-	-
	**Negative** effect of age							
left middle temporal gyrus	-	-	-	-	5.23	<.0001	31	.019
left inferior parietal lobule	-	-	-	-	4.69	<.0001	115	.012
left inferior frontal gyrus	-	-	-	-	4.64	<.0001	29	.042

Note: No exact MNI coordinates given due to the use of custom-made template during normalization process. k_E_ = extent threshold.

Within the Global_FWE_ ROI, individual LIs showed a tendency to correlate with age (*r* = -.217, *p* = .096). Outside the Global_FWE_ ROI, no such correlation was detectable (*r* = -.140, *p* = .201). In the frontal ROI, no additional positive or negative effects of age were detected. 

### 3.4: Performance effects

**Figure 4:**
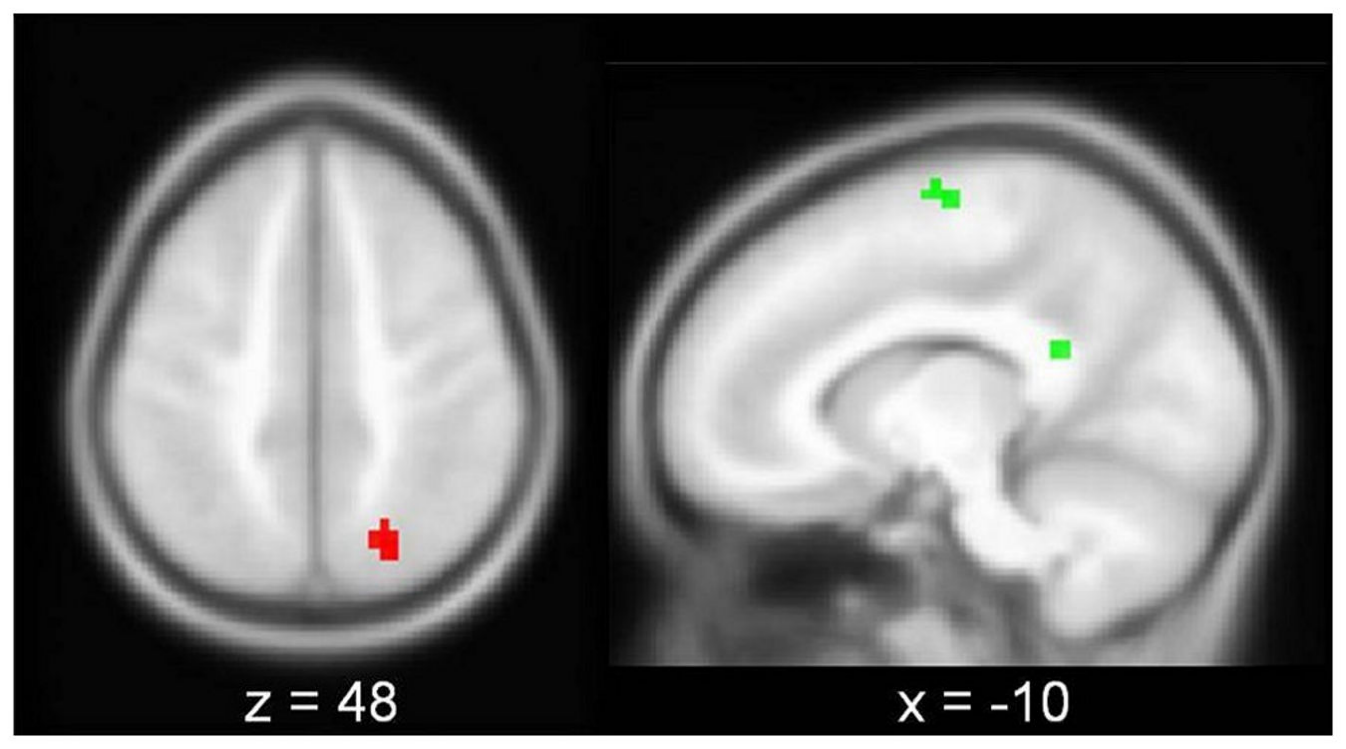
Performance-related activation increases within (red) and decreases outside (green) the global task-related network (Global_FWE_) overlaid on sections (MNI coordinate of slice) of the custom group T1 template. All clusters within ROIs at uncorrected p < .001 and extent threshold k > 15.

Effects of hit rate were detected within and outside Global_FWE_ (Figure 4, Table 2). Inside the ROI, hit rate correlated positively with activation in right superior parietal lobe. Outside the ROI, hit rate correlated with activation in medial and inferior occipital cortex. Within Global_FWE_, hit rate did not correlate negatively with activation. Outside the ROI, however, a negative effect of hit rate was detected in left precentral gyrus and in the vicinity of left posterior corpus callosum/ posterior cingulate. Lateralization of activation was not correlated to hit rate, neither within (*r* = -.045, *p* = .394), nor outside the Global_FWE_ ROI (*r* = -.020, *p* = .452). 

**Table 2:** Hit-rate-related fMRI activation.

	Inside Global_FWE_				Outside Global_FWE_			
	Peak level		Cluster level		Peak level		Cluster level	
	T	p_uncorr_	k_E_	p_uncorr_	T	p_uncorr_	k_E_	p_uncorr_
	**Positive** effect of hit rate							
medial occipital cortex	-	-	-	-	4.76	<.0001	100	.021
inferior occipital cortex	-	-	-	-	4.05	<.0001	47	.033
right superior parietal lobule	3.87	<.0001	19	.252	-	-	-	-
	**Negative** effect of hit rate							
left precentral gyrus	-	-	-	-	4.44	<.0001	21	.090
posterior corpus callosum	-	-	-	-	4.17	<.0001	24	.133

Note: No exact MNI coordinates given due to the use of custom-made template during normalization process. k_E_ = extent threshold.

In the frontal ROI, no additional positive or negative effects of performance were detected. 

## Discussion

Studying the effects of age and performance on fMRI activation during a complex visual search task, our main finding was an increase of activation with age in bilateral occipital cortex and right superior parietal lobule. Thus, the older were our study participants, the stronger was their BOLD response during the visual search task within core regions of the network for complex visual search. In addition, activation decreased with age in a left-hemispheric network of inferior frontal, middle temporal, and superior parietal cortex. Thus, the younger our participants, the more did they recruit left-hemispheric brain regions outside the core network for complex visual search. This corroborates our hypothesis (H1). Hypothesis (H2), which predicted a performance-related increase of lateralization within the same regions, received only little support: Age and performance were correlated only in the right superior parietal lobule in the ROI analyses. However, the correlations of lateralization index with age or performance within the whole network did not reach significance. Hypothesis (H3) could not be corroborated, since we did not find effects of age or performance within the prefrontal cortex. 

### 4.1: fMRI activation during complex visual search

The comparison of a complex visual search task with a very simple pattern-matching task highlights a broad network of predominantly posterior brain regions. Considering the various processes involved in performing complex visual search, involvement of a range of brain regions was expected. The overall group activation pattern included large portions of both the superior and the inferior parietal lobe. This is well in line with the task requirements: First of all, subjects needed to shift their visual attention (ideally systematically) from focus to focus in the images, and thus should engage their superior parietal lobe, intraparietal sulcus, frontal eye fields [67], and the medial superior frontal sulcus [68]. These processes needed for complex visual search are also necessary to solve the embedded figures task (EFT). The EFT has been associated with left-lateralized activation in inferior and superior parietal lobules and ventral premotor cortex in fMRI experiments [44]. In our study, the activation pattern is bilateral and slightly right-lateralized. Since the widely-reported hemispheric dissociation for local vs. global visual processing (e.g., 69) seems to be on the one hand subtle in nature and on the other hand strongly dependent on the experimental conditions [70], this is not surprising. In natural life conditions, attention shifts are usually entailing saccades. To keep the task as simple as possible, we allowed eye movements and thus expect saccades in our subjects, which are mediated predominantly by the frontal eye fields [8]. While the activation we see in the middle frontal gyrus may seem too far lateral to reflect FEF activity, it still seems well compatible with Paus' regional range of +/- 11 mm in left-right, +/‑ 5 mm in anterior-posterior, and +/- 5 mm in inferior-superior direction [8]. 

### 4.2: Age effects

The analysis of the effects of age on activation in the main visual search network reveals an age-related increase of activation in occipital and superior parietal cortices. At the same time, activation outside the main visual search network, namely in left inferior parietal cortex, left middle temporal cortex and left inferior frontal cortex decreases with age. Our assumption that frontal regions “join task execution” successively during adolescence was not corroborated, since we did not find any age effects in the prefrontal ROI. Thus, our data did not provide evidence for the frontalization-with-age hypothesis as seen in other tasks which rely more heavily on executive functions [37]. The reverse effects of age on activation within and outside the network for complex visual search, however, indicate that older subjects more exclusively recruit its core components while younger children involved additional brain regions to solve the task. We propose that the younger children used verbal strategies (as likely reflected by left inferior frontal cortex activation), which is in line with previous findings for the EFT [45]. The left inferior parietal cortex activation in younger vs. older subjects is similar to the effect observed in mental rotation tasks [39]. In these tasks, children are assumed to employ piecemeal-like vs. holistic processing strategies to solve the task [39]. Given the complexity of the search task we used, the same could apply for our paradigm. While the lateralization indices do not correlate significantly with age in the ROIs explored, an effect of age is observed only in the right superior parietal lobule. This observation would again be in line with a growing efficacy of brain networks with age [35], in our case the predominantly right-hemispheric posterior visual processing network.

Two alternative explanations for the result of a more wide-spread activation in the younger children could be that 1) the normalization process was more accurate for older children, and 2) younger children displayed more motion than older children. However, both explanations seem very unlikely given 1) our use of a custom-made age-adjusted template [60], and 2) the introduction of specific movement parameters into the individual single-subject statistics on the first level [63]." 

### 4.3: Performance effects

As children and adolescents grow older, they get more proficient in many complex visual processing tasks. Thus, it is difficult to disentangle the effect of performance on brain activation from the effect of age. Including age as covariate into the analyses regarding performance and vice versa is an attempt to take this problem into consideration. While not a perfect solution and likely being too conservative due to removing shared variance from either analysis, it seems a defensible approach. Within the visual search network, performance impacts activation in the right superior parietal lobule – the same region that shows an increased activation with increasing age. Thus, one could assume that better performance is correlated with a more mature network in complex visual search. On the other hand, we found that subjects with low hit rates engaged more regions outside the visual exploration network, such as left precentral gyrus and in the vicinity of the posterior cingulate. It is difficult to interpret this activation pattern. The most probable explanation here is one of response inhibition which could be reflected in an increased contralateral motor-activation (since the subjects responded with their left hands) and error detection. Given the effects of normalization and smoothing, the exact localization of group activation peaks is difficult to accomplish [71]. Based on the literature, we tentatively propose that our posterior activation cluster could reflect posterior cingulate cortex activation which has been correlated with unsuccessful trials in cognitive control tasks [72].

One reason for the very small effects of performance on the activation pattern might be the way of performance operationalization. It should be mentioned that “successful target trial” was defined as a button press in response to a target trial. Since all trials were presented for a fixed duration of six seconds, misses were composed of those trials where no target was detected within this specified time (but could have been detected during a longer time interval). Of note, a low hit rate in this context is not equivalent to guessing or lack of attention, but to lack of target detection during the time provided. The very high hit rate during the control condition indicates a good overall task adherence, so that it can be assumed that subjects have principally attended to the tasks [27]. Hit rate is therefore a mixture of rapidity and quality of visual exploration and thus probably not the most sensitive measure. In a self-paced design, these two components could have been disentangled, which might allow for a more comprehensive description of performance effects. Also, the inclusion of subjects with a wider range of cognitive abilities might have allowed us to detect these regions with a higher level of statistical reliability.

## Conclusion

In a complex visual search task we found evidence for an age-related shift from diffuse to focal activation of a predominantly right-hemispheric visuospatial occipito-parietal network. This means that younger age was associated with a more widespread and more bilateral activation pattern, probably reflecting alternative, presumably verbal and/or piecemeal-like vs. holistic strategies for task performance. Better performance correlated with increased activation of right superior parietal lobule, thus presumably reflecting a more mature network in high-performers. No specific effects of age or performance were detected in prefrontal cortex. We believe our results are encouraging with respect to the feasibility to disentangle age and performance during physiological maturation, using fMRI. 

## References

[1] Kravitz DJ, Saleem KS, Baker CI, Mishkin M (2011) A new neural framework for visuospatial processing. Nat Rev Neurosci 12: 217-230. doi:10.1038/nrn3008. PubMed: 21415848. 21415848 PMC3388718

[2] Mishkin M, Ungerleider LG (1982) Contribution of striate inputs to the visuospatial functions of parieto-preoccipital cortex in monkeys. Behav Brain Res 6: 57-77. doi:10.1016/0166-4328(82)90081-X. PubMed: 7126325. 7126325

[3] Hayakawa T, Fujimaki N, Imaruoka T (2006) Temporal characteristics of neural activity related to target detection during visual search. NeuroImage 33: 296-306. doi:10.1016/j.neuroimage.2006.06.034. PubMed: 16919970. 16919970

[4] Shipp S (2011) Interhemispheric integration in visual search. Neuropsychologia 49: 2630-2647. doi:10.1016/j.neuropsychologia.2011.05.011. PubMed: 21640738. 21640738 PMC3149659

[5] Nobre AC, Coull JT, Walsh V, Frith CD (2003) Brain Activations during Visual Search: Contributions of Search Efficiency versus Feature Binding. NeuroImage 18: 91-103. doi:10.1006/nimg.2002.1329. PubMed: 12507447. 12507447

[6] Muggleton NG, Juan C-H, Cowey A, Walsh V (2003) Human Frontal Eye Fields and Visual. Search - Journal of Neurophysiology 89: 3340-3343. doi:10.1152/jn.01086.2002. 12783960

[7] Leonards U, Sunaert S, Van Hecke P, Orban GA (2000) Attention Mechanisms in Visual Search—An fMRI Study. J Cogn Neurosci 12: 61-75. doi:10.1162/089892900564073. PubMed: 11506648. 11506648

[8] Paus T (1996) Location and function of the human frontal eye-field: A selective review. Neuropsychologia 34: 475-483. doi:10.1016/0028-3932(95)00134-4. PubMed: 8736560. 8736560

[9] Corbetta M, Miezin FM, Shulman GL, Petersen SE (1993) A PET study of visuospatial attention. Journal of Neuroscience 13: 1202-1226. PubMed: 8441008. 8441008 10.1523/JNEUROSCI.13-03-01202.1993 PMC6576604

[10] Corbetta M, Akbudak E, Conturo TE, Snyder AZ, Ollinger JM et al. (1998) A Common Network of Functional Areas for Attention and Eye Movements. Neuron 21: 761-773. doi:10.1016/S0896-6273(00)80593-0. PubMed: 9808463. 9808463

[11] Grosbras M-H, Laird AR, Paus T (2005) Cortical regions involved in eye movements, shifts of attention, and gaze perception. Hum Brain Mapp 25: 140-154. doi:10.1002/hbm.20145. PubMed: 15846814. 15846814 PMC6871707

[12] Muggleton NG, Juan C-H, Cowey A, Walsh V (2003) Human Frontal Eye Fields and Visual. Search - Journal of Neurophysiology 89: 3340-3343. doi:10.1152/jn.01086.2002. 12783960

[13] Coull JT, Frith CD (1998) Differential Activation of Right Superior Parietal Cortex and Intraparietal Sulcus by Spatial and Nonspatial Attention. NeuroImage 8: 176-187. doi:10.1006/nimg.1998.0354. PubMed: 9740760. 9740760

[14] Culham JC, Kanwisher NG (2001) Neuroimaging of cognitive functions in human parietal cortex. Curr Opin Neurobiol 11: 157-163. doi:10.1016/S0959-4388(00)00191-4. PubMed: 11301234. 11301234

[15] Leonards U, Sunaert S, Van Hecke P, Orban GA (2000) Attention Mechanisms in Visual Search—An fMRI Study. J Cogn Neurosci 12: 61-75. doi:10.1162/089892900564073. PubMed: 11506648. 11506648

[16] Nobre AC, Coull JT, Walsh V, Frith CD (2003) Brain Activations during Visual Search: Contributions of Search Efficiency versus Feature Binding. NeuroImage 18: 91-103. doi:10.1006/nimg.2002.1329. PubMed: 12507447. 12507447

[17] Petersen SE, Corbetta M, Miezin FM, Shulman GL (1994) PET studies of parietal involvement in spatial attention: Comparison of different task types. Canadian Journal of Experimental Psychology /Revue canadienne de psychologie expérimentale 48: 319-338 10.1037/1196-1961.48.2.319 8069288

[18] Wojciulik E, Kanwisher N (1999) The Generality of Parietal Involvement in Visual Attention. Neuron 23: 747-764. doi:10.1016/S0896-6273(01)80033-7. PubMed: 10482241. 10482241

[19] Silk TJ, Bellgrove MA, Wrafter P, Mattingley JB, Cunnington R (2010) Spatial working memory and spatial attention rely on common neural processes in the intraparietal sulcus. NeuroImage 53: 718-724. doi:10.1016/j.neuroimage.2010.06.068. PubMed: 20615473. 20615473

[20] Posner MI, Walker JA, Friedrich FJ, Rafal RD (1984) Effects of parietal injury on covert orienting of attention. Journal of Neuroscience 4: 1863-1874. PubMed: 6737043. 6737043 10.1523/JNEUROSCI.04-07-01863.1984 PMC6564871

[21] Wolfe JM, Cave KR, Franzel SL (1989) Guided search: An alternative to the feature integration model for visual search. J Exp Psychol Hum Percept Perform 15: 419-433. PubMed: 2527952. 2527952 10.1037//0096-1523.15.3.419

[22] Himmelbach M, Erb M, Karnath H-O (2006) Exploring the visual world: The neural substrate of spatial orienting. NeuroImage 32: 1747-1759. doi:10.1016/j.neuroimage.2006.04.221. PubMed: 16806986. 16806986

[23] Anderson EJ, Mannan SK, Husain M, Rees G, Sumner P et al. (2007) Involvement of prefrontal cortex in visual search. Experimental Brain Research 180: 289-302. doi:10.1007/s00221-007-0860-0. PubMed: 17310377. 17310377

[24] Bunge SA, Wright SB (2007) Neurodevelopmental changes in working memory and cognitive control. Curr Opin Neurobiol 17: 243-250. doi:10.1016/j.conb.2007.02.005. PubMed: 17321127. 17321127

[25] Makino Y, Yokosawa K, Takeda Y, Kumada T (2004) Visual search and memory search engage extensive overlapping cerebral cortices: an fMRI study. NeuroImage 23: 525-533. doi:10.1016/j.neuroimage.2004.06.026. PubMed: 15488401. 15488401

[26] Karnath HO, Rorden C (2012) The anatomy of spatial neglect. Neuropsychologia 50: 1010-1017. doi:10.1016/j.neuropsychologia.2011.06.027. PubMed: 21756924. 21756924 PMC3348466

[27] Ebner K, Lidzba K, Hauser TK, Wilke M (2011) Assessing language and visuospatial functions with one task: a "dual use" approach to performing fMRI in children. NeuroImage 58: 923-929. doi:10.1016/j.neuroimage.2011.06.048. PubMed: 21726649. 21726649

[28] Everts R, Lidzba K, Wilke M, Kiefer C, Mordasini M et al. (2009) Strengthening of laterality of verbal and visuospatial functions during childhood and adolescence. Hum Brain Mapp 30: 473-483. doi:10.1002/hbm.20523. PubMed: 18219619. 18219619 PMC6871185

[29] Carpenter PA, Just MA, Keller TA, Eddy W, Thulborn K (1999) Graded Functional Activation in the Visuospatial System with the Amount of Task Demand. J Cogn Neurosci 11: 9-24. doi:10.1162/089892999563210. PubMed: 9950711. 9950711

[30] Witkin HA (1950) Individual differences in ease of perception of embedded figures. J Pers 19: 1-15. doi:10.1111/j.1467-6494.1950.tb01084.x. PubMed: 14795367. 14795367

[31] Walter E, Dassonville P (2011) Activation in a frontoparietal cortical network underlies individual differences in the performance of an embedded figures task. PLOS ONE 6: e20742. doi:10.1371/journal.pone.0020742. PubMed: 21799729. 21799729 PMC3140479

[32] Smith SE, Chatterjee A (2008) Visuospatial attention in children. Arch Neurol 65: 1284-1288. PubMed: 18852341. 18852341 10.1001/archneur.65.10.1284

[33] Akshoomoff NA, Stiles J (1995) Developmental trends in visuospatial analysis and planning: I. Copying a complex figure. Neuropsychology 9: 364-377. doi:10.1037/0894-4105.9.3.364.

[34] Pezdek K, Roman Z, Sobolik KG (1986) Spatial memory for objects and words. Journal of Experimental Psychology. Learning, Memory, and Cognition 12: 530-537.

[35] Durston S, Casey BJ (2006) What have we learned about cognitive development from neuroimaging? Neuropsychologia 44: 2149-2157. doi:10.1016/j.neuropsychologia.2005.10.010. PubMed: 16303150. 16303150

[36] Casey BJ, Giedd JN, Thomas KM (2000) Structural and functional brain development and its relation to cognitive development. Biol Psychol 54: 241-257. doi:10.1016/S0301-0511(00)00058-2. PubMed: 11035225. 11035225

[37] Rubia K, Overmeyer S, Taylor E, Brammer M, Williams SCR et al. (2000) Functional frontalisation with age: mapping neurodevelopmental trajectories with fMRI. Neurosci Biobehav Rev 24: 13-19. PubMed: 10654655. 10654655 10.1016/s0149-7634(99)00055-x

[38] Friston KJ, Williams S, Howard R, Frackowiak RS, Turner R (1996) Movement-related effects in fMRI time-series. Magn Reson Med 35: 346-355. doi:10.1002/mrm.1910350312. PubMed: 8699946. 8699946

[39] Jansen-Osmann P, Heil M ( January 182007) Developmental aspects of parietal hemispheric asymmetry during mental rotation. Neuroreport January 18: 175-178. PubMed: 17301685. 10.1097/WNR.0b013e328010ff6b 17301685

[40] Bryce D, Szũcs D, Soltész F, Whitebread D (2011) The development of inhibitory control: An averaged and single-trial Lateralized Readiness Potential study. NeuroImage 57: 671-685. doi:10.1016/j.neuroimage.2010.12.006. PubMed: 21146618. 21146618

[41] Rubia K, Smith AB, Woolley J, Nosarti C, Heyman I et al. (2006) Progressive increase of frontostriatal brain activation from childhood to adulthood during event-related tasks of cognitive control. Hum Brain Mapp 27: 973-993. doi:10.1002/hbm.20237. PubMed: 16683265. 16683265 PMC6871373

[42] Rubia K, Hyde Z, Halari R, Giampietro V, Smith A (2010) Effects of age and sex on developmental neural networks of visual-spatial attention allocation. NeuroImage 51: 817-827. doi:10.1016/j.neuroimage.2010.02.058. PubMed: 20188841. 20188841

[43] Manjaly ZM, Bruning N, Neufang S, Stephan KE, Brieber S et al. (2007) Neurophysiological correlates of relatively enhanced local visual search in autistic adolescents. NeuroImage 35: 283-291. doi:10.1016/j.neuroimage.2006.11.036. PubMed: 17240169. 17240169 PMC2644454

[44] Manjaly ZM, Marshall JC, Stephan KE, Gurd JM, Zilles K et al. (2003) In search of the hidden: an fMRI study with implications for the study of patients with autism and with acquired brain injury. NeuroImage 19: 674-683. doi:10.1016/S1053-8119(03)00095-8. PubMed: 12880798. 12880798

[45] Lee PS, Foss-Feig J, Henderson JG, Kenworthy LE, Gilotty L et al. (2007) Atypical neural substrates of Embedded Figures Task performance in children with Autism Spectrum Disorder. NeuroImage 38: 184-193. doi:10.1016/j.neuroimage.2007.07.013. PubMed: 17707658. 17707658 PMC2084060

[46] Klingberg T, Forssberg H, Westerberg H (2002) Increased Brain Activity in Frontal and Parietal Cortex Underlies the Development of Visuospatial Working Memory Capacity during Childhood. J Cogn Neurosci 14: 1-10. doi:10.1162/089892902317205276. PubMed: 11798382. 11798382

[47] Kwon H, Reiss AL, Menon V (2002) Neural basis of protracted developmental changes in visuo-spatial working memory. Proc Natl Acad Sci U S A 99: 13336-13341. doi:10.1073/pnas.162486399. PubMed: 12244209. 12244209 PMC130634

[48] Scherf KS, Sweeney JA, Luna B (2006) Brain Basis of Developmental Change in Visuospatial Working. Memory - Journal of Cognitive Neuroscience 18: 1045-1058. doi:10.1162/jocn.2006.18.7.1045. 16839280

[49] Groen MA, Whitehouse AJ, Badcock NA, Bishop DV (2011) Where were those rabbits? A new paradigm to determine cerebral lateralisation of visuospatial memory function in children. Neuropsychologia 49: 3265-3271. doi:10.1016/j.neuropsychologia.2011.07.031. PubMed: 21843539. 21843539 PMC3198251

[50] Marsh R, Zhu H, Schultz RT, Quackenbush G, Royal J et al. (2006) A developmental fMRI study of self-regulatory control. Hum Brain Mapp 27: 848-863. doi:10.1002/hbm.20225. PubMed: 16421886. 16421886 PMC2292452

[51] Szaflarski JP, Altaye M, Rajagopal A, Eaton K, Meng X et al. (2012) A 10-year longitudinal fMRI study of narrative comprehension in children and adolescents. NeuroImage 63: 1188-1195. doi:10.1016/j.neuroimage.2012.08.049. PubMed: 22951258. 22951258 PMC3476849

[52] Rubia K, Smith AB, Woolley J, Nosarti C, Heyman I et al. (2006) Progressive increase of frontostriatal brain activation from childhood to adulthood during event-related tasks of cognitive control. Hum Brain Mapp 27: 973-993. doi:10.1002/hbm.20237. PubMed: 16683265. 16683265 PMC6871373

[53] Crone EA, Donohue SE, Honomichl R, Wendelken C, Bunge SA (2006) Brain regions mediating flexible rule use during development. J Neurosci 26: 11239-11247. doi:10.1523/JNEUROSCI.2165-06.2006. PubMed: 17065463. 17065463 PMC6674662

[54] Dekker T, Mareschal D, Sereno MI, Johnson MH (2011) Dorsal and ventral stream activation and object recognition performance in school-age children. NeuroImage 57: 659-670. doi:10.1016/j.neuroimage.2010.11.005. PubMed: 21056677. 21056677

[55] Oldfield RC (1971) The assessment and analysis of handedness: The Edinburgh inventory. Neuropsychologia 9: 97-113. doi:10.1016/0028-3932(71)90067-4. PubMed: 5146491. 5146491

[56] Lidzba K, Staudt M, Wilke M, Grodd W, Krägeloh-Mann I (2006) Lesion-induced right-hemispheric language and organization of nonverbal functions. Neuroreport 17: 929-933. doi:10.1097/01.wnr.0000221841.12632.d6. PubMed: 16738490. 16738490

[57] Rey A (1941) L'examen psychologique dans les cas d'encephalopathie traumatique. Archives of Psychology (Chicago) 28: 286-340.

[58] Andersson JL, Hutton C, Ashburner J, Turner R, Friston K (2001) Modeling geometric deformations in EPI time series. NeuroImage 13: 903-919. doi:10.1016/S1053-8119(01)92245-1. PubMed: 11304086. 11304086

[59] Ashburner J, Friston KJ (2005) Unified segmentation. NeuroImage 26: 839-851. doi:10.1016/j.neuroimage.2005.02.018. PubMed: 15955494. 15955494

[60] Wilke M, Holland SK, Altaye M, Gaser C (2008) Template-O-Matic: a toolbox for creating customized pediatric templates. NeuroImage 41: 903-913. doi:10.1016/j.neuroimage.2008.02.056. PubMed: 18424084. 18424084

[61] Macey PM, Macey KE, Kumar R, Harper RM (2004) A method for removal of global effects from fMRI time series. NeuroImage 22: 360-366. doi:10.1016/j.neuroimage.2003.12.042. PubMed: 15110027. 15110027

[62] Friston KJ, Frith CD, Frackowiak RS, Turner R (1995) Characterizing dynamic brain responses with fMRI: a multivariate approach. NeuroImage 2: 166-172. doi:10.1006/nimg.1995.1019. PubMed: 9343599. 9343599

[63] Wilke M (2012) An alternative approach towards assessing and accounting for individual motion in fMRI timeseries. NeuroImage 59: 2062-2072. doi:10.1016/j.neuroimage.2011.10.043. PubMed: 22036679. 22036679

[64] Wilke M, Lidzba K (2007) LI-tool: A new toolbox to assess lateralization in functional MR-data. J Neurosci Methods 163: 128-136. doi:10.1016/j.jneumeth.2007.01.026. PubMed: 17386945. 17386945

[65] Wilke M, Schmithorst VJ (2006) A combined bootstrap/histogram analysis approach for computing a lateralization index from neuroimaging data. NeuroImage 33: 522-530. doi:10.1016/j.neuroimage.2006.07.010. PubMed: 16938470. 16938470

[66] Hammers A, Allom R, Koepp MJ, Free SL, Myers R et al. (2003) Three-dimensional maximum probability atlas of the human brain, with particular reference to the temporal lobe. Hum Brain Mapp 19: 224-247. doi:10.1002/hbm.10123. PubMed: 12874777. 12874777 PMC6871794

[67] Tamber-Rosenau BJ, Esterman M, Chiu Y-C, Yantis S (2011) Cortical Mechanisms of Cognitive Control for Shifting Attention in Vision and Working. Memory - Journal of Cognitive Neuroscience 23: 2905-2919. doi:10.1162/jocn.2011.21608. 21291314 PMC3158824

[68] Nagahama Y, Okada T, Katsumi Y, Hayashi T, Yamauchi H et al. (1999) Transient Neural Activity in the Medial Superior Frontal Gyrus and Precuneus Time Locked with Attention Shift between Object Features. NeuroImage 10: 193-199. doi:10.1006/nimg.1999.0451. PubMed: 10417251. 10417251

[69] Fink GR, Halligan PW (1996) Where in the brain does visual attention select the forest and the trees? Nature 382: 626–628. doi:10.1038/382626a0. PubMed: 8757132. 8757132

[70] Yovel G, Yovel I, Levy J (2001) Hemispheric asymmetries for global and local visual perception: Effects of stimulus and task factors. J Exp Psychol Hum Percept Perform 27: 1369-1385. PubMed: 11766931. 11766931

[71] Reimold M, Slifstein M, Heinz A, Mueller-Schauenburg W, Bares R (2006) Effect of spatial smoothing on t-maps: arguments for going back from t-maps to masked contrast images. J Cereb Blood Flow Metab 26: 751-759. doi:10.1038/sj.jcbfm.9600231. PubMed: 16208316. 16208316

[72] Rubia K, Smith AB, Taylor E, Brammer M (2007) Linear age-correlated functional development of right inferior fronto-striato-cerebellar networks during response inhibition and anterior cingulate during error-related processes. Hum Brain Mapp 28: 1163-1177. doi:10.1002/hbm.20347. PubMed: 17538951. 17538951 PMC6871440

